# Understanding efficacy-safety balance of biologics in moderate-to-severe pediatric psoriasis

**DOI:** 10.3389/fmed.2022.944208

**Published:** 2022-09-26

**Authors:** Klervi Golhen, Carolyn Winskill, Martin Theiler, Michael Buettcher, Yu-Hsin Yeh, Nancy Zhang, Tatjana Welzel, Marc Pfister

**Affiliations:** ^1^Pediatric Pharmacology and Pharmacometrics, University Children’s Hospital Basel (UKBB), University of Basel, Basel, Switzerland; ^2^Integrated Drug Development, Certara LP, Princeton, NJ, United States; ^3^Pediatric Skin Center, Dermatology Department, University Children’s Hospital Zurich, Zurich, Switzerland; ^4^Pediatric Infectious Diseases Unit, Pediatric Department, Children’s Hospital – Lucerne Cantonal Hospital, Lucerne, Switzerland; ^5^Pediatric Rheumatology, University Children’s Hospital Basel (UKBB), University of Basel, Basel, Switzerland

**Keywords:** systematic literature review, meta-analysis, biologics, efficacy, safety, pediatric, psoriasis

## Abstract

**Background:**

Psoriasis is a chronic immune-mediated inflammatory skin disease affecting both adults and children. To better understand the efficacy-safety profile of biologics in children with moderate-to-severe psoriasis, this study aimed to analyze efficacy and safety data of randomized controlled trials (RCTs) performed in pediatric psoriasis and to compare efficacy outcomes in children with those in adults.

**Methods:**

RCTs investigating biologics in children with moderate-to-severe psoriasis were identified in a systematic literature review. PASI75/90 treatment responses at weeks 11/12 were analyzed comparing biologics with control arms. Serious adverse events (SAEs) were analyzed at the end of each study. Efficacy data from RCTs in adults with psoriasis were selected for the same biologics. Risk ratios (RR) of selected RCTs were pooled together in a statistical random effects model using the inverse variance method.

**Results:**

For children, there were 1 etanercept, 2 secukinumab, 1 ixekizumab and 1 ustekinumab placebo-controlled RCTs and 1 adalimumab RCT using methotrexate as reference arm at weeks 11/12. For adults, out of 263 RCTs, 7 adalimumab and 15 etanercept (TNF inhibitors) and 4 ixekizumab and 12 ustekinumab (IL-17 and IL-12/23 inhibitors) RCTs reported PASI75/90 efficacy responses at weeks 11/12. Regarding efficacy, all biologics showed improved PASI responses over control arms. RRs ranges were 2.02–7.45 in PASI75 and 4.10–14.50 in PASI90. The highest PASI75 responses were seen for ustekinumab 0.375 mg/kg (RR = 7.25, 95% CI 2.83–18.58) and ustekinumab 0.75 mg/kg (RR = 7.45, 95% CI 2.91–19.06) in the CADMUS study. The highest PASI90 response was seen for ixekizumab (RR = 14.50, 95% CI 4.82–43.58) in the IXORA-PEDS study. SAE incidences in pediatric and adult arms with biologics were 0 to 3% except for a pediatric arm with adalimumab 0.40 mg/kg (8%). For adults, pooled RR also showed improved PASI responses over placebo for all biologics, with highest PASI75 response observed for ixekizumab (pooled RR = 16.18, 95% CI 11.83–22.14).

**Conclusion:**

Both adults and children with psoriasis show superior efficacy with biologics compared to control arms after 3 months of treatment with SAE incidences in the low percentages. Additional longer-term clinical studies are warranted to fully understand the overall efficacy-safety profile of biologics in children with moderate-to-severe psoriasis.

## Introduction

Psoriasis is a chronic immune-mediated systemic disease with the skin as the major affected organ presenting with well-demarcated pink-to-erythematous plaques with overlying hyperkeratotic plaques. The onset may be during child- or adulthood. The worldwide prevalence is estimated at 0.51 to 11.3% in adults and 0 to 1.37% in children (1). Chronic plaque psoriasis is the most common form in children, typically involving the face and scalp. Psoriasis is often associated with serious comorbidities such as cardiovascular disease, metabolic syndrome, chronic kidney disease, arthritis, psychosocial effects, and inflammatory bowel disease (2, 3). Risk factors may include environmental (skin trauma, infections, medications, and psychological stress) and genetic [human leukocyte antigen (HLA) type Cw6 (*PSORS1*) or *CARD14* mutation] factors (4). Psoriasis is driven by the innate and adaptive immune systems, partly characterized by the chronic activation of T-helper cells (Th17) and secretion of proinflammatory cytokines such as interleukin 17 (IL-17) and tumor necrosis factor-α (TNF-α) in response to IL-23 (5). Diagnosis is based on the clinical features and most patients do not need a skin biopsy for histopathological evaluation. The Physician’s Global Assessment of Disease Activity (PGA) is a 6-point scale used to measure the severity of disease at the time of the evaluation. Severity and extent of psoriasis disease activity can be measured in children and adults with the Psoriasis Area and Severity Index (PASI). Psoriasis is grouped into “mild-to-moderate” (less than 10% of body surface area involved) and “moderate-to-severe” (more than 10% of body surface area involved) plaque psoriasis. The “moderate-to-severe” group, approximately 10 to 20% of the pediatric age group, often requires phototherapy and/or systemic treatment. Without effective treatment, psoriasis results in decreased psychosocial health and reduced quality of life (6). Several indices exist to assess the impact of psoriasis on the health-related quality of life (HRQOL) in children. The Children’s Dermatology Life Quality Index (CDLQI) is a 10-item questionnaire for measuring HRQOL in children aged from 4 to 16 years (7).

In the past, established therapeutic management included topical agents, phototherapy, conventional systemic treatments (e.g., methotrexate, ciclosporin, and acitretin), and biologics such as TNF inhibitors (8). Recent breakthroughs in the understanding of psoriasis pathogenesis have resulted in the use of IL-17 and IL-12/23 targeted therapies. The approval of IL-17 and IL-12/23 inhibitors has revolutionized treatment of moderate-to-severe plaque psoriasis in adults and most compounds have recently been approved for use in children aged 6 years and older (9). These new treatment options have significantly facilitated treatment of children with moderate-to-severe psoriasis (10). Given the requirement for potentially lifelong treatment, an excellent safety profile is paramount, especially when treating children. Currently, there are only a limited number of studies comparing different biologics in the pediatric population, making decisions on what biologic to use difficult. Finding the preferred initial biologic for children is challenging and has to be based on factors of safety, patient comorbidities, frequency of dosing and drug availability and licensing in the respective country.

This study aimed to (i) summarize and analyze available efficacy and safety data of randomized controlled trials (RCTs) performed in pediatric psoriasis patients, (ii) compare published treatment outcomes with those in adults, and (iii) provide an overview of current psoriasis treatment strategies in children and adults to (iv) better understand the efficacy-safety balance of biologics with the goal of supporting clinicians in their therapeutic decisions in clinical practice, and those developing new treatment options for children with moderate-to-severe psoriasis.

## Methods

This systematic review was conducted based on the Cochrane Handbook for Systematic Reviews of Interventions and reporting items in the PRISMA statement (11, 12) and focused on comparing risk ratios (RRs) of PASI75 or PASI90 (efficacy endpoints) and incidence of adverse events (AEs) of interest (safety endpoints) between RCTs in adults and children with psoriasis treated with biologics. It should be noted that the typical timepoint for efficacy and safety evaluation for pivotal RCTs in pediatric drug development and approval is 12 weeks. Therefore, a majority of reported pivotal clinical RCTs in children with moderate-to-severe psoriasis investigated efficacy and safety endpoints 3 months after treatment start. For this reason we focused our systematic review and meta-analysis on one key timepoint: drug-related effects on efficacy and safety at week 11/12 of treatment.

### Literature search and selection of trials

**FIGURE 1:**
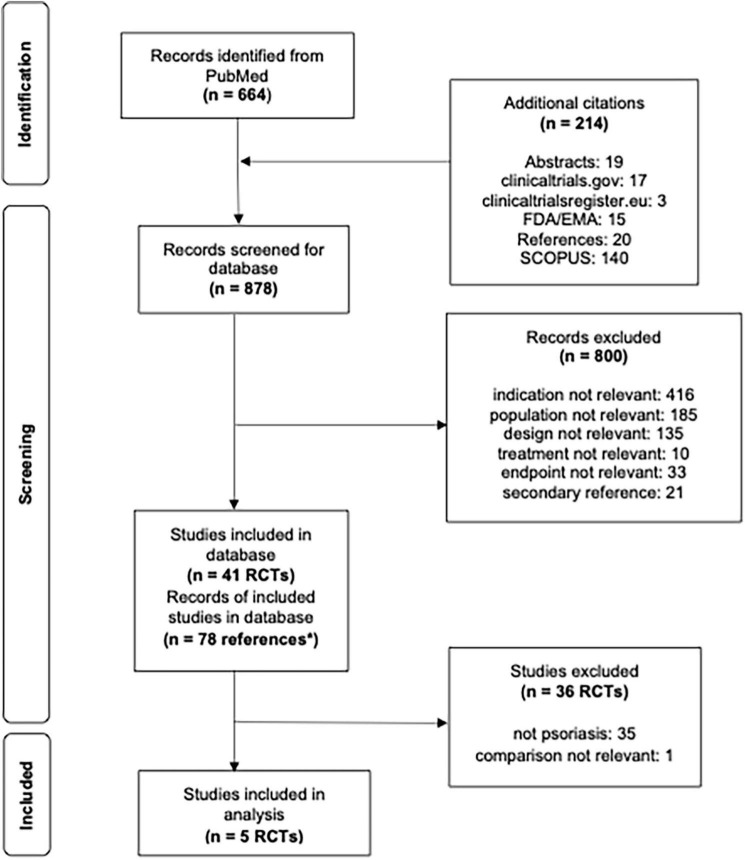
Flowsheet literature review and pediatric study selection for analysis. *n = 78 correspond to multiple references to a single study, i.e., more than one journal article, clinicaltrials.gov and/or regulatory documents.

Randomized controlled trials for all pediatric rheumatology patients were identified in a systematic literature search initially conducted on July 26, 2020 in MEDLINE, ClinicalTrials.gov and the EU Clinical Trials Register with a sample size of ≥5 children with pediatric inflammatory rheumatic disease (PiRD), including moderate-to-severe psoriasis (PiRD) aged ≤20 years and treated with predefined biologics (13). On February 3, 2022 this literature search was updated in line with the previously described review protocol to ensure that the most recent publications on pediatric moderate-to-severe psoriasis are included (Figure 1) (13). An additional search was conducted in the SCOPUS database using the same search terms used for MEDLINE but this did not yield any additional publications of relevance. When multiple references for a single study (e.g., more than one journal article^1^, regulatory documents) were available, data from one study were pulled from all available sources. Identified RCTs fulfilling the following inclusion criteria were included in the analysis: (i) population: plaque psoriasis; (ii) treatment: abatacept, adalimumab, anakinra, baricitinib, belimumab, brodalumab, canakinumab, certolizumab, etanercept, golimumab, guselkumab, infliximab, ixekizumab, rilonacept, risankizumab, rituximab, sarilumab, secukinumab, tildrakizumab, tocilizumab, tofacitinib, upadacitinib, or ustekinumab; (iii) control: placebo, no treatment, or conventional systemic therapies including methotrexate, ciclosporin, or acitretin; (iv) outcomes: PASI75 or PASI90 responses; (v) time point: 3 months after treatment start (at week 11/12). Funnel plots of all included studies were performed to assess publication bias (Supplementary Figure 2).

Randomized controlled trials for adult plaque psoriasis patients that met similar criteria as the RCTs for pediatric psoriasis were similarly identified from an existing up-to-date clinical outcomes database (Certara Psoriasis CODEx database) as of October 11, 2021. The search for the adult psoriasis RCTs used the following search terms: (psoria*[TIAB]) AND (list of drugs) AND (randomized controlled trial[publication type] OR (randomized [TIAB] OR randomized [TIAB])) NOT case reports[publication type] NOT review[publication type] NOT comment[publication type] NOT letter[publication type] NOT rats[MeSH] AND humans[MeSH]. Studies strictly in psoriatic arthritis patients were excluded. To facilitate analysis, other exclusion criteria were applied that differed from the selection of pediatric studies: (i) control: placebo only; and (ii) sample size of ≥ 20 patients.

It should be noted that only data from RCTs were considered for systematic review and meta-analyses. Observational studies and review articles were excluded.

### Efficacy and safety outcomes datasets

#### Data collection

Aggregate (summary)-level data was extracted for each selected RCT. Study design, baseline demographic and clinical characteristics such as location, patient population, sample size, age criteria, and treatment, as well as efficacy and safety data were captured. In addition, detailed dosing information from published RCTs in children and adults with psoriasis were tabulated.

#### Efficacy outcomes data

The Psoriasis Area and Severity Index responses PASI75 and PASI90 at weeks 11 or 12 (i.e., after 3 months of treatment) were used to describe efficacy for both adults and children. In cases of duplicate data within a study, data from the intent-to-treat (ITT) population was selected over per-protocol or completed populations and imputed data was selected over non-imputed data. PGA ≤ 1 and CDLQI ≤ 1 responses after 3 months of treatment were also extracted for exploratory analysis.

#### Safety outcomes data

Each RCT for adult and pediatric psoriasis patients included in the systematic literature review was screened for safety data at the end of induction treatment (i.e., 12 or 16 weeks). Safety data was selected according to hierarchical inclusion criteria to ensure each study arm had only one data point: (1) overall population with all grades of AEs, (2) stratified population with all grades of AEs (if data was reported separately for different subgroups, like regions of an arm instead of the arm overall), and (3) overall population with non-serious AEs. Safety data of interest included (i) overall AEs, (ii) serious adverse events (SAEs), (iii) overall infections, (iv) serious infections, (v) upper respiratory tract infections (URTIs), (vi) gastroenteritis, (vii) autoimmune reactions, and (viii) dermatologic AEs. To maintain consistency across studies, only the proportion or number of patients with AEs was captured. Rate data (events/patient-year or total number of events) was not captured.

### Systematic review and analyses for efficacy and safety

#### Efficacy outcomes

For the pediatric data, raw proportions (%) in each study arm for PASI75 and PASI90 responses 3 months after treatment initiation were recorded or calculated as the number of subjects with response divided by the total number of subjects evaluable for response. RR were calculated as percentage of response in biologics arm divided by percentage of response in control arm. Further, 95% confidence intervals (CIs) for RRs were computed utilizing the exact method. All CIs that did not include 1 indicated significant effects. RRs of PASI75 and PASI90 3 months after treatment initiation were visualized using forest plots. The pooled RR for each outcome was calculated utilizing the DerSimonian-Laird method with a random effects model. Heterogeneity between studies was assessed using I^2^ (the proportion of variability between studies due to heterogeneity). Heterogeneity was defined by the following *I*^2^ thresholds: no heterogeneity *I*^2^ = 0%, low *I*^2^ < 30%, moderate 30 ≤ *I*^2^ ≤ 59%, and high *I*^2^ ≥ 60%. Since the meta-analysis for each PASI outcome included fewer than 10 studies, statistical significance of heterogeneity was assessed as *p* < 0.10. Meta-regression was also not performed due to the number of studies. Raw proportions (%) of participants achieving a PGA score of 0 or 1 and a CDLQI score of 0 or 1 3 months after treatment initiation were also recorded for each study. For the adult data, RR for all four efficacy outcomes for each distinct biologic were grouped and pooled together in a statistical random effects model using the inverse variance method.

#### Safety outcomes

The incidence of each AE of interest was summarized descriptively for each arm at the end of each study in addition to calculating the risk difference (percentage of AEs in biologic arm - percentage of AEs in control arm). For the adult data, the number of patients with the AE and the number of patients evaluable for safety analysis were combined across arms evaluating the same treatment of interest (i.e., all adalimumab data were pooled together and all placebo data were pooled together for the same group of studies).

Dosing information from approved biologics in children and adults was summarized in Table 5.

**TABLE 1:** Overview of adult psoriasis studies used for comparison against pediatric psoriasis studies.

Drug class	Drug	Study	Registry ID	Dose	Phase	Ndrug	Nplacebo	Ntotal	PASI75	PASI90	PGA	DLQI	AE
TNF inhibitor	adalimumab	CHAMPION	NCT00235820	40 mg	3	324	159	483	yes	yes	yes	no	yes
		M02-528	NCT00645814	40 mg	2	96	52	148	yes	no	no	no	yes
		M04-688	NCT00338754	40 mg; 80 mg	2/3	246	92	338	yes	yes	no	no	no
		M13-606	NCT01646073	40 mg	3	1352	348	1700	yes	yes	yes	yes	yes
		REVEAL	NCT00237887	40 mg	3	2442	1194	3636	yes	yes	yes	no	yes
		VOYAGE 1	NCT02207231, EudraCT2014-000719-15	40 mg	3	668	348	1016	yes	yes	yes	no	yes
		X-PLORE	NCT01483599, EudraCT2011-001066-17	40 mg	2	129	126	255	yes	yes	yes	no	yes
						5257	2319	7576					
	certolizumab	C87040	NCT00245765, EudraCT2005-002141-39	200 mg; 400 mg	2	468	236	704	yes	yes	yes	yes	yes
		CIMPACT	NCT02346240, EudraCT2014-003492-36	200 mg; 400 mg	3	996	171	1167	yes	yes	yes	no	yes
		CIMPASI-1	NCT02326298	400| 200 mg; 400 mg	3	549	153	702	yes	yes	yes	no	yes
		CIMPASI-2	NCT02326272	400| 200 mg; 400 mg	3	534	147	681	yes	yes	yes	no	yes
						2547	707	3254					
	etanercept	20030117	NCT00111449	50 mg	3	622	618	1240	yes	yes	no	no	yes
		20030211	NCT00078819	0.8 mg/kg	3	318	315	633	yes	yes	yes	no	yes
		A3921080	NCT01241591	50 mg	3	1344	432	1776	yes	yes	yes	yes	yes
		CIMPACT	NCT02346240, EudraCT2014-003492-36	50 mg	3	510	171	681	yes	yes	yes	no	yes
		FIXTURE	NCT01358578	50 mg	3	978	978	1956	yes	yes	yes	no	yes
		Gottlieb AB 2003		25 mg	2	114	110	224	yes	yes	no	no	no
		Leonardi CL 2003		25 mg; 50 mg	3	1512	504	2016	yes	yes	yes	no	yes
		M10-114	NCT00691964	50 mg	3	423	204	627	yes	yes	yes	no	yes
		M10-315	NCT00710580	50 mg	3	417	216	633	yes	yes	yes	no	yes
		Papp KA 2005		25 mg; 50 mg	3	1221	612	1833	yes	yes	yes	no	yes
		reSURFACE 2	NCT01729754	50 mg	3	939	468	1407	yes	yes	yes	yes	yes
		UNCOVER-2	NCT01597245	50 mg	3	1432	672	2104	yes	yes	yes	yes	yes
		UNCOVER-3	NCT01646177	50 mg	3	1528	772	2300	yes	yes	yes	yes	yes
		van de Kerkhof PC 2008		50 mg	2	384	184	568	yes	yes	yes	yes	yes
						11742	6256	17998					
IL-17 inhibitor	brodalumab	20060279	NCT00867100	140 mg; 350 mg; 700 mg	1	63	15	78	yes	yes	yes	no	yes
		20090062	NCT00975637, EudraCT2009-013539-39	70 mg; 140 mg; 210 mg; 280 mg	2	640	152	792	yes	yes	yes	yes	yes
		4827-002	NCT01748539	70 mg; 140 mg; 210 mg	2	339	114	453	yes	yes	yes	no	yes
		4827-KR001	NCT02982005	210 mg/day	3	120	66	186	yes	yes	yes	no	yes
		AMAGINE-1	NCT01708590	140 mg; 210 mg	3	1323	660	1983	yes	yes	yes	no	yes
		AMAGINE-2	NCT01708603	140 mg; 210 mg	3	3666	927	4593	yes	yes	yes	no	yes
		AMAGINE-3	NCT01708629	140 mg; 210 mg	3	3759	945	4704	yes	yes	yes	no	yes
						9910	2879	12789					
	ixekizumab	I1F-MC-RHAJ	NCT01107457	10 mg; 25 mg; 75 mg; 150 mg	2	345	81	426	yes	yes	yes	no	no
		UNCOVER-1	NCT01474512	80 mg	3	2595	1293	3888	yes	yes	yes	no	yes
		UNCOVER-2	NCT01597245	80 mg	3	2792	672	3464	yes	yes	yes	yes	yes
		UNCOVER-3	NCT01646177	80 mg	3	3084	772	3856	yes	yes	yes	yes	yes
						8816	2818	11634					
	secukinumab	ALLURE	NCT02748863	300 mg; 0| 300 mg	3b	497	213	710	yes	yes	yes	no	yes
		CAIN457A2102	NCT00669916	3 mg/kg	2	36	36	72	yes	yes	no	no	yes
		CAIN457A2211	NCT00941031	150 mg	2	1348	268	1616	yes	yes	yes	yes	yes
		CAIN457A2212	NCT00805480	3 mg/kg; 10 mg/kg	2	270	30	300	yes	yes	yes	no	no
		CAIN457A2220	NCT01071252	25 mg; 75 mg; 150 mg	2	233	66	299	yes	yes	yes	no	yes
		CAIN457A2318	NCT03066609, EudraCT 2016-000524-25	150 mg; 300 mg	3b	1224	405	1629	yes	yes	yes	no	no
		CAIN457AUS02	NCT02690701	300 mg	4	138	135	273	yes	yes	yes	no	no
		CARIMA	NCT02559622, EudraCT 2013-002266-40	300 mg	3	96	98	194	yes	yes	no	no	yes
		CCJM112 × 2101_multiple dose	NCT01828086, EudraCT 2012-004507-12	150 mg	1	12	12	24	yes	yes	no	no	no
		ERASURE	NCT01365455, EudraCT2010-023512-13	300 mg; 150 mg	3	1960	992	2952	yes	yes	yes	yes	yes
		FEATURE	NCT01555125	300 mg; 150 mg	3	472	236	708	yes	yes	yes	yes	yes
		FIXTURE	NCT01358578	150 mg; 300 mg	3	1962	978	2940	yes	yes	yes	no	yes
		JUNCTURE	NCT01636687	300 mg; 150 mg	3	484	244	728	yes	yes	yes	yes	yes
		ObePso-S	NCT03055494	300 mg	4	54	28	82	no	yes	no	no	yes
						8786	3741	12527					
IL-12/23 inhibitor	ustekinumab	AMAGINE-2	NCT01708603	45,90 mg	3	900	927	1827	yes	yes	yes	no	yes
		AMAGINE-3	NCT01708629	45,90 mg	3	939	945	1884	yes	yes	yes	no	yes
		CR005416	NCT00320216	45 mg; 90 mg	2	768	192	960	yes	yes	yes	no	yes
		CR015166	NCT00723528	45 mg; 90 mg	3	504	184	688	yes	yes	yes	yes	no
		LOTUS	NCT01008995	45 mg	3	480	486	966	yes	yes	yes	no	yes
		PEARL	NCT00747344	45 mg	3	244	240	484	yes	yes	yes	yes	yes
		PHOENIX1	NCT00267969, EudraCT2005-003529-15	45 mg; 90 mg	3	2044	1020	3064	yes	yes	yes	yes	yes
		PHOENIX2	NCT00307437, EudraCT2005-003530-17	45 mg; 90 mg	3	3280	1640	4920	yes	yes	yes	yes	yes
		UltIMMa-1	NCT02684370, EudraCT2014-005117-23	45,90 mg	3	300	306	606	yes	yes	yes	no	yes
		UltIMMa-2	NCT02684357, EudraCT2015-003622-13	45,90 mg	3	297	294	591	yes	yes	yes	no	yes
		VIP-U	NCT02187172	45,90 mg	4	44	42	86	yes	yes	no	no	no
		Zhou 2020		45 mg	3	11	13	24	yes	no	no	no	no
						9811	6289	16100					
IL-23 inhibitor	guselkumab	CR103833	NCT02325219	50 mg; 100 mg	3	256	128	384	yes	yes	yes	no	yes
		new (ORION)	NCT02905331, EudraCT2016-002022-37	100 mg	3	186	48	234	yes	yes	yes	no	yes
		VOYAGE 1	NCT02207231, EudraCT2014-000719-15	100 mg	3	329	174	503	yes	yes	yes	no	yes
		VOYAGE 2_withdrawal	NCT02207244, EudraCT2014-000720-18	100 mg	3	579	546	1125	yes	yes	yes	no	no
		X-PLORE	NCT01483599, EudraCT2011-001066-17	5 mg; 15 mg; 50 mg; 100 mg; 200 mg	2	624	126	750	yes	yes	yes	no	yes
						1974	1022	2996					
	risankizumab	1311.1	NCT01577550	0.01, 0.05, 0.25, 1, 3, 5 (iv); 0.25, 1 (sc) mg/kg	1	132	24	156	yes	yes	yes	no	no
		UltIMMa-1	NCT02684370, EudraCT2014-005117-23	150 mg	3	912	306	1218	yes	yes	yes	no	yes
		UltIMMa-2	NCT02684357, EudraCT2015-003622-13	150 mg	3	882	294	1176	yes	yes	yes	no	yes
						1926	624	2550					
	tildrakizumab	MK-3222-003	NCT01225731	5 mg; 25 mg; 100 mg; 200 mg	2b	309	46	355	yes	no	no	no	yes
		reSURFACE 1	NCT01722331	100 mg; 200 mg	3	2468	620	3088	yes	yes	yes	yes	yes
		reSURFACE 2	NCT01729754	100 mg; 200 mg	3	2484	624	3108	yes	yes	yes	yes	yes
						5261	1290	6551					

AE, adverse event; DLQI, Dermatology Life Quality Index; ID, identification number; Ndrug, number of patients receiving the drug; Nplacebo, number of patients receiving the placebo; Ntotal, total number of patients; PASI, Psoriasis Area and Severity Index; PGA, Physician Global Assessment; sc, subcutaneous; iv, intravenous.

**TABLE 2:** Overview of PASI75/90 data at 3 months in RCTs in children with psoriasis.

Drug class	Drug	Study	Number of included patients		PASI75&	PASI90&
			Biologics study arm	Control study arm		
TNF inhibitor	adalimumab LD#	M04-717	39	37	44%| 22%	28%| 3%
TNF inhibitor	adalimumab HD#	M04-717	38	37	60%| 22%	29%| 3%
TNF inhibitor	etanercept	20030211	106	105	57%| 11%	27%| 7%
IL-17 inhibitor	ixekizumab	IXORA-PEDS	115	56	89%| 25%	78%| 5%
IL-12/23 inhibitor	ustekinumab LD	CADMUS	37	37	78%| 11%	54%| 5%
IL-12/23 inhibitor	ustekinumab HD	CADMUS	36	37	81%| 11%	61%| 5%
IL-17 inhibitor	secukinumab LD	CAIN457A2310	40	41	80%| 15%	73%| 2%
IL-17 inhibitor	secukinumab HD	CAIN457A2310	40	41	78%| 15%	68%| 2%
TNF inhibitor	etanercept	CAIN457A2310	41	41	63%| 15%	29%| 2%

HD, high dose; LD, low dose; PASI, Psoriasis Area and Severity Index; & proportion of patients in biologics study arm | proportion of patients in control study arm with response; # compared against methotrexate monotherapy instead of placebo.

**TABLE 3:** Overview of PGA and CDLQI data at 3 months in RCTs in children with psoriasis.

Drug class	Drug	Study	PGA ≤ 1&	PGA ≤ 1 RR	CDLQI ≤ 1&	CDLQI ≤ 1 RR
TNF inhibitor	adalimumab LD#	M04-717	31%|19%	1.63 (0.72–3.69)		
TNF inhibitor	adalimumab HD#	M04-717	47%|19%	2.51 (1.19–5.29)		
TNF inhibitor	etanercept	20030211	53%|13%	3.96 (2.36–6.66)		
IL-17 inhibitor	ixekizumab	IXORA-PEDS	81%|11%	7.56 (3.53–16.20)	64%|23%	2.77 (1.69–4.55)
IL-12/23 inhibitor	ustekinumab LD	CADMUS	68%|5%	12.50 (3.19–49.01)	39%|13%	2.90 (1.05–8.00)
IL-12/23 inhibitor	ustekinumab HD	CADMUS	69%|5%	12.85 (3.28–50.32)	57%|13%	4.25 (1.62–11.15)
IL-17 inhibitor	secukinumab LD	CAIN457A2310	70%|5%		45%|15%	
IL-17 inhibitor	secukinumab HD	CAIN457A2310	60%|5%		50%|15%	
TNF inhibitor	etanercept	CAIN457A2310	34%|5%		37%|15%	

CDLQI, Children’s Dermatology Life Quality Index; HD, high dose; LD, low dose; PGA, Physician Global Assessment. & proportion of patients in biologics arm | proportion of patients in control arm with response. # compared against methotrexate monotherapy instead of placebo.

**TABLE 4:** Overview of adverse events of interest in RCTs in children with psoriasis.

Drug	Study	Biologics arm	Control arm	Time (weeks)	Allergic or autoimmune reactions %	Dermatologic reactions %	Respiratory infections %	Gastrointestinal or hepatic reactions %
adalimumab LD#	M04-717	39	37	16	allergic reaction (1| 2), injection site reaction (3| 3)	skin papilloma* (1| 0), tinea versicolor (1| 0), impetigo (0| 1)	acute sinusitis (1| 0), pharyngitis (0| 1), sinusitis (0| 1), tonsillitis (0| 1), tracheitis (0| 1), URTI severe (0| 1), URTI (4| 6), nasopharyngitis (10| 7), rhinitis (1| 1), viral URTI (1| 1)	gastroenteritis (0| 3), gastrointestinal infection (1| 0)
adalimumab HD#	M04-717	38	37	16	allergic reaction (0| 2), injection site reaction (4| 3)	impetigo (0| 1)	acute tonsillitis (1| 0), pharyngitis (0| 1), sinusitis (0| 1), URTI severe (0| 1), URTI (2| 6), nasopharyngitis (8| 7), pharyngitis streptococcal (1| 0), rhinitis (3| 1), tonsillitis (0| 1), tracheitis (0| 1), viral URTI (1| 1)	gastroenteritis (2| 3)
etanercept	20030211	106	105	12	injection site reaction (7| 5)	skin infection* (1| 1), skin papilloma* (2| 0)	pharyngitis* (2| 4), sinusitis* (1| 1), URTI* (18| 12), pharyngitis streptococcal* (3| 1), nasopharyngitis* (8| 9)	gastroenteritis* (6| 0), gastroenteritis viral* (2| 3)
ixekizumab	IXORA-PEDS	115	56	12	injection site reaction (14| 1)	impetigo (1| 0), folliculitis (1| 0)	pharyngitis (2| 0), URTI (6| 4), nasopharyngitis (13| 4), pharyngitis streptococcal (2| 0), pharyngotonsillitis (1| 0), tonsilitis (1| 2), viral URTI (2| 0)	
ustekinumab LD	CADMUS	37	37	12			bronchitis* (0| 1), pharyngitis* (3| 0), URTI* (1| 2), nasopharyngitis* (5| 10)	gastroenteritis* (1| 1)
ustekinumab HD	CADMUS	36	37	12			bronchitis* (1| 1), pharyngitis* (1| 0), pharyngitis streptococcal* (1| 0), URTI* (3| 2), nasopharyngitis* (1| 10)	gastroenteritis* (0| 1)
secukinumab LD	CAIN457A2310	40	41	12	injection site reaction (2| 2)			
secukinumab HD	CAIN457A2310	40	41	12	injection site reaction (0| 2)			
etanercept	CAIN457A2310	41	41	12	injection site reaction (3| 2)			

AE, adverse event; HD, high dose; LD, low dose; LRTI, lower respiratory tract infection; RTI, respiratory tract infection; URTI, upper respiratory tract infection. # compared against methotrexate monotherapy instead of placebo; *specified as non-serious AE. % number of patients in biologics arm | number of patients in control arm.

**TABLE 5:** Overview of approved biologics in adult versus pediatric psoriasis.

Drug class	Drug	Adult approval	Adult approved dose	Pediatric approval	Pediatric approved dose
TNF inhibitor	adalimumab	2007 (FDA), 2008 (EMA)	40 mg q2w (FDA/EMA)	2015 (EMA, > = 4 years)	20 mg (<30 kg) or 40 mg (> = 30 kg) q2w (EMA)
	certolizumab pegol	2018 (FDA/EMA)	400 mg 0, 2, 4, 200 mg q2w (FDA/EMA)		
	etanercept	2004 (FDA/EMA)	50 mg biw/qw (FDA), 25 mg biw or 50 mg qw (EMA)	2008 (EMA, > = 6 years), 2016 (FDA, > = 4 years)	0.8 mg/kg qw (FDA/EMA)
	infliximab	2005 (EMA), 2006 (FDA)	5 mg/kg 0, 2, 6, q8w (FDA/EMA)		
IL-12/23 inhibitor	ustekinumab	2008 (EMA), 2009 (FDA)	45 or 90 mg (>100 kg) 0, 4, q12w (FDA/EMA)	2015 (EMA, > = 6 years), 2017 (FDA, > = 6 years)	0.75 mg/kg (<60 kg), 45 mg (60–100 kg) or 90 mg (>100 kg) 0, 4, q12w (FDA/EMA)
IL-17 inhibitor	brodalumab	2017 (FDA/EMA)	210 mg 0, 1, 2, q2w (FDA/EMA)		
	ixekizumab	2016 (FDA/EMA)	80 mg q2w/q4w (FDA/EMA)	2020 (FDA/EMA both > = 6 years)	20 mg (<25 kg), 40 mg (25–50 kg), or 80 mg (>50 kg) q4w (FDA), 40 (25–50 kg) or 80 mg (>50 kg) q4w (EMA)
	secukinumab	2014 (EMA), 2015 (FDA)	300 mg q4w (FDA/EMA)	2020 (EMA, > = 6 years), 2021 (FDA, > = 6 years)	75 (<50 kg) or 150 mg (> = 50 kg) q4w (FDA/EMA)
IL-23 inhibitor	guselkumab	2017 (FDA/EMA)	100 mg 0, 4, q8w (FDA/EMA)		
	risankizumab	2019 (FDA/EMA)	150 mg 0, 4, q12w (FDA/EMA)		
	tildakizumab	2018 (FDA/EMA)	100 mg 0, 4, q12w (FDA/EMA)		

EMA, European Medicines; Agency; FDA, Food and Drug Administration; NA, not available; qXw, every X weeks.

#### Publication bias and software package

Publication bias was assessed using visual inspection of the funnel plots; asymmetry of the funnel plots was assessed using Begg’s test (rank correlation method) and Egger’s test (linear regression method). Statistical significance for publication bias was assessed as *p* < 0.05. Meta-analyses were performed using the “meta” package in R (version 4.0.3). All forest plots and other graphs were generated using RStudio (version 1.2.5042).

### Overview of approved doses of biologics in pediatric and adult psoriasis

A comprehensive overview of currently available dose information from approved biologics in children and adults is provided.

## Results

In this section we summarize results from the performed literature search, data collection, and systematic literature review for efficacy and safety outcomes in pediatric and adult RCTs. We also provide an overview of approved doses of biologics in pediatric psoriasis and compare those with dosing approaches in adults with psoriasis.

### Literature search and selection of trials

Six out of a total of 41 PiRD studies were conducted in pediatric psoriasis patients. Two studies investigated secukinumab [IL-17 inhibitor], CAIN457A2310 (NCT02471144) (14) and CAIN457A2311 (NCT03668613) (15, 16). The other four studies investigated other biologics (M04-717 (NCT01251614) [adalimumab, TNF inhibitor] (16), 20030211 (NCT00078819) [etanercept, TNF inhibitor] (17–19), IXORA-PEDS (NCT03073200) [ixekizumab, IL-17 inhibitor] (20), CADMUS (NCT01090427) [ustekinumab, IL-12/23 inhibitor]) (21). No studies were identified in pediatric patients treated with JAK inhibitors. All the inclusion criteria of the pediatric studies required the patients to be candidates for systemic therapy; to be poorly controlled by topical therapy; to have a history of psoriasis for at least 6 months; a PASI of at least 12 (with the exception of a PASI score of at least 20 for adalimumab, secukinumab, and ixekizumab in countries where etanercept was approved); a PGA of at least 3; body surface area (BSA) involvement at screening and baseline of at least 10% (except adalimumab with at least 20% BSA involved). Only the adalimumab trial required heliotherapy or phototherapy to have failed, be contraindicated or not tolerated; in no other trial previous treatment with a conventional systemic or phototherapy was required. All six studies reported PASI75 and PASI90 at weeks 11 or 12. The CAIN457A2311 secukinumab study was excluded from analysis as the comparison was out of scope (secukinumab high dose versus secukinumab low dose). The five remaining studies reported PGA ≤ 1 at week 12 and three studies reported CDLQI ≤ 1 at week 12. The comparator arm for the adalimumab study was methotrexate while the other four studies were placebo controlled (Figure 1).

Out of a total of 263 adult studies available in the Certara CODEx database, there were 7 adalimumab (22–28), 7 brodalumab (29–34), 4 certolizumab (35–37), 14 etanercept (17, 36, 38–49), 4 ixekizumab (46, 50, 51), 5 guselkumab (27, 28, 52, 53), 3 risankizumab (54, 55), 14 secukinumab (42, 56–63), 3 tildrakizumab (47, 64), and 12 ustekinumab (34, 55, 65–71) placebo-controlled RCTs that reported PASI75/PASI90 treatment responses at weeks 11 or 12 (Table 1). To be consistent with the pediatric studies, only monotherapy arms were selected for analysis. Inclusion criteria for pediatric studies compared to adult studies seem to be comparable or slightly more stringent regarding severity (PASI) and less stringent regarding pre-treatment with systemic treatments and phototherapy, given that these treatments are not licensed for the studied population. Overall, it is not possible to exclude that the adult population had slightly more severe disease at inclusion, given that patients had to be refractory to standard care including systemic treatment or phototherapy.

### Systematic review for efficacy outcomes

In this section we report the key efficacy endpoints PASI75 and PASI90, Physician’s Global Assessment of Disease Activity (PGA) ≤ 1 and Children’s Dermatology Life Quality Index (CDLQI) ≤ 1 in children with psoriasis, and PASI75, PASI90, PGA, and DLQI in adults with psoriasis.

#### PASI75 and PASI90 in children

**FIGURE 2:**
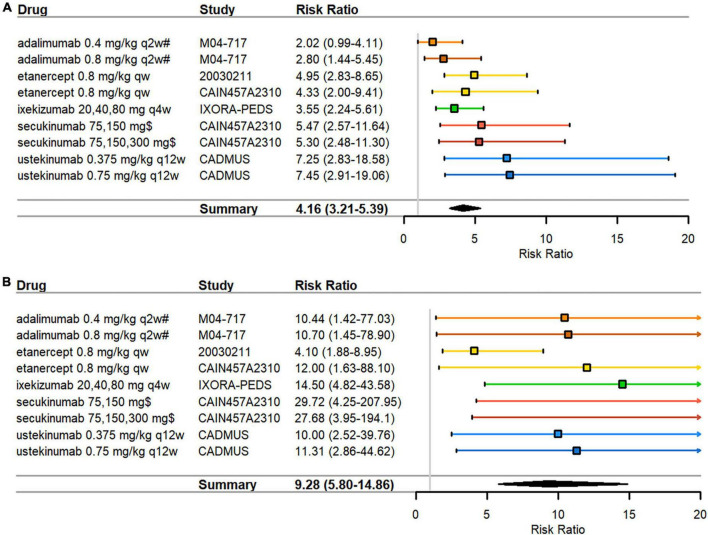
Pediatric psoriasis RCTs that reported efficacy data at 3 months. **(A)** PASI75 **(B)** PASI90 RRs—mean represented by the square—were calculated as percent response in biologics arm divided by percent response in control arm. Overall effect estimate is represented by the diamond, with the width showing the CIs for the overall estimated effect estimate. Further, 95% CIs for RRs—represented by the whiskers—were computed utilizing the exact method. All CIs that did not include 1 indicated significant effects. Experimental treatment is preferred when RR > 1. PASI, Psoriasis Area and Severity Index; qw, every week; q2w, every 2 weeks; q4w, every 4 weeks; q12w, every 12 weeks; RCT, randomized controlled trial; RR, risk ratio. $ dosed at weeks 1, 2, 3, 4, and every 4 weeks thereafter; # compared against methotrexate monotherapy instead of placebo.

All biologics arms in the pediatric psoriasis RCTs indicated significant treatment effects in PASI responses over control arms (Table 2). RRs ranged from 2.02–7.45 in PASI75 (Figure 2A) and 4.10–29.72 in PASI90 (Figure 2B). Disease severity was moderate to severe in all RCTs at inclusion with the exception of the CADMUS study (severe). The lowest PASI75 treatment response was seen for adalimumab and the lowest PASI90 treatment response in a study arm with etanercept (both TNF inhibitors). The highest PASI75 treatment responses were seen for ustekinumab 0.375 mg/kg (RR = 7.25, 95% CI 2.83–18.58) and ustekinumab 0.75 mg/kg (RR = 7.45, 95% CI 2.91–19.06) compared to placebo in the CADMUS study (Figure 2A). The highest PASI90 treatment responses were seen for ixekizumab (RR = 14.50, 95% CI 4.82–43.58) in the IXORA-PEDS study and for secukinumab (RR = 29.72 for low dose and RR = 27.68 for high dose) in the CAIN457A2310 study (Figure 2B). The pooled RR was 4.16 (95% CI 3.21–5.39) for PASI75 (Figure 2A) and 9.28 (95% CI 5.80–14.86) for PASI90 (Figure 2B), indicating that arms with biologics are significantly superior compared to control arms. There was low heterogeneity in the meta-analysis of both PASI outcomes (*I*^2^ = 21% and *p* = 0.26 for PASI75; *I*^2^ = 3% and *p* = 0.41 for PASI90) (data not shown).

Funnel plot asymmetry was significant by Egger’s test for PASI90 (*p* = 0.017) (Supplementary Figure 2B) but not significant for PASI75 (*p* = 0.243) (Supplementary Figure 2A). Funnel plot asymmetry was not significant by Begg’s test for PASI75 (*p* = 0.144) or PASI90 (*p* = 0.677). Due to the low number of studies, results must be interpreted with caution as tests for publication bias are underpowered when there are fewer than 10 studies in an analysis.

#### PASI75 and PASI90 in adults

The pooled RR for the corresponding adult psoriasis data (Supplementary Table 1) also showed improved PASI responses over placebo for all biologics, with the highest PASI75 response observed for ixekizumab (pooled RR = 16.18, 95% CI 11.83–22.14) and secukinumab (pooled RR = 15.35, 95% CI 12.49–18.86). Ixekizumab and secukinumab also showed the highest PASI90 treatment effects compared to placebo (Supplementary Table 1). Of the biologics approved in adults but still undergoing investigation in children, guselkumab had the largest PASI75 RR compared to the other biologics (pooled RR = 23.02, 95% CI 7.70–68.83) (Supplementary Table 1).

#### Physician’s Global Assessment of Disease Activity in children

The proportion of patients achieving a PGA of 0 or 1 after 3 months of treatment was higher in arms with biologics than in placebo or standard of care (SOC) arms. In the IXORA-PEDS study, 81% of patients achieved a PGA score of 0 or 1 at week 12 in the ixekizumab arm versus 11% in the placebo arm (RR = 7.56, 95% CI 3.53–16.20) (Table 3). Ustekinumab (68% for low dose and 69% for high dose) and secukinumab low dose (70%) reported the highest percentages of patients achieving a PGA score of 0 or 1 after 3 months of treatment.

#### Physician’s Global Assessment of Disease Activity in adults

Pooled RRs for PGA of 0 or 1 indicated significant treatment effects for biologics compared to placebo in adult studies (Supplementary Table 2). The highest PGA ≤ 1 response was observed for certolizumab (pooled RR = 30.47, 95% CI 11.48–80.86) and the lowest response observed for etanercept (pooled RR = 7.74, 95% CI 5.83–10.27). Pooled RRs were also high for the IL-17 inhibitors (brodalumab, ixekizumab, secukinumab).

#### Children’s Dermatology Life Quality Index in children

Absolute change and percentage of improvement from baseline in CDLQI at week 12 was higher in biologic arms than in placebo or SOC arms. Ixekizumab (64%) and ustekinumab high dose (57%) reported the highest percentages of patients achieving CDLQI ≤ 1 after 3 months of treatment (Table 3). Compared to placebo responses, ustekinumab showed the highest treatment response (RR = 4.25, 95% CI 1.62–11.15). In the 20030211 study, CDLQI improved by 52% from baseline in the etanercept arm versus 18% in the placebo arm (data not shown). In the M04-717 study, CDLQI scores decreased by 4.9 points in the adalimumab low-dose arm, 6.6 points in the adalimumab high-dose arm, and 5 points in the methotrexate arm (data not shown).

#### Dermatology Life Quality Index in adults

Available data indicates that biologics in adult RCTs showed improved responses over placebo (Supplementary Table 2). No DLQI endpoints were reported for guselkumab or risankizumab studies, and only one study was only available for other biologics. Ustekinumab had the highest treatment response compared to placebo (pooled RR = 12.23, 95% CI 8.72–17.15). Pooled RRs were also high for all IL-17 inhibitors.

### Systematic review for safety outcomes

In this section we report SAEs, adverse events, and anti-drug antibodies in children and adults with psoriasis.

#### Serious adverse events in children

**FIGURE 3:**
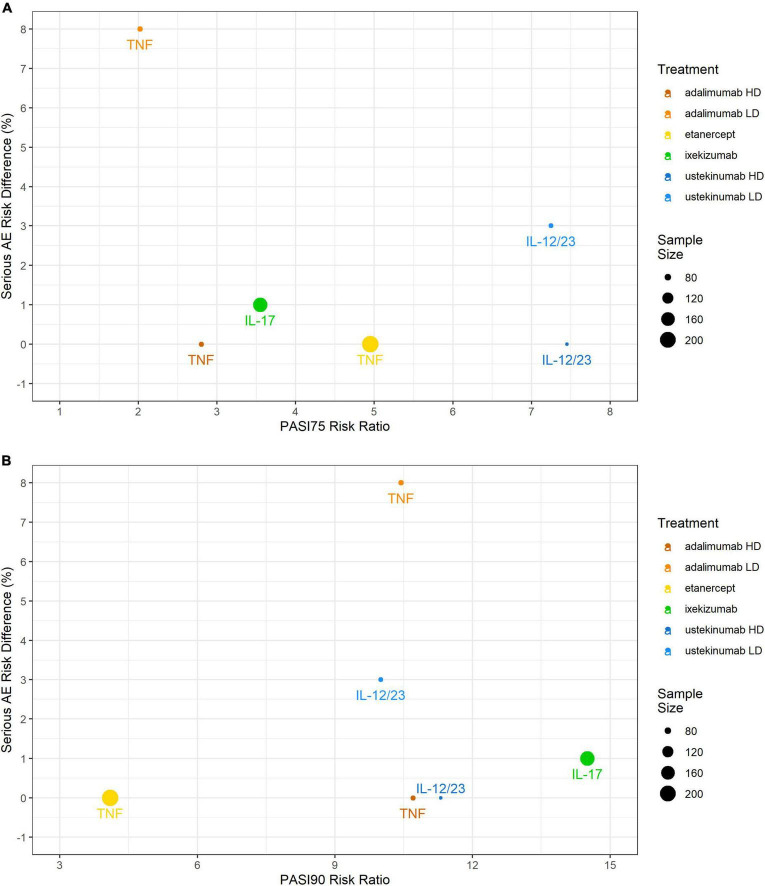
Comparing efficacy (PASI75 and PASI90 RRs) against safety (RD) in the pediatric psoriasis RCTs that reported both outcomes. **(A)** PASI75 RR versus SAEs RD; **(B)** PASI90 RR versus SAEs RD. PASI, Psoriasis Area and Severity Index; RCT, randomized controlled trial; RD, risk difference; SAE, serious adverse event.

Serious adverse events incidences were low with the IL-17 inhibitor ixekizumab (1%), the IL-12/23 inhibitor ustekinumab (3%), and the TNF inhibitor etanercept (0%). With the TNF inhibitor adalimumab, compared to the 0.4 mg/kg study arm (8%; gastrointestinal infection, hand fracture, agitation), while it was low in the 0.8 mg/kg study arm (0%). No SAEs were reported in any of the pediatric control study arms. No serious infections were reported except for the study arm with adalimumab 0.4 mg/kg (3%) (Figure 3).

#### Serious adverse events in adults

Serious adverse event incidences in adults were low in all study arms (up to 3%) with an RR of 1.08 in IL-17 inhibitors brodalumab, ixekizumab and secukinumab, and an RR of 1.09 in the IL-12/23 inhibitor ustekinumab (Supplementary Table 3).

#### Adverse events in children

**FIGURE 4:**
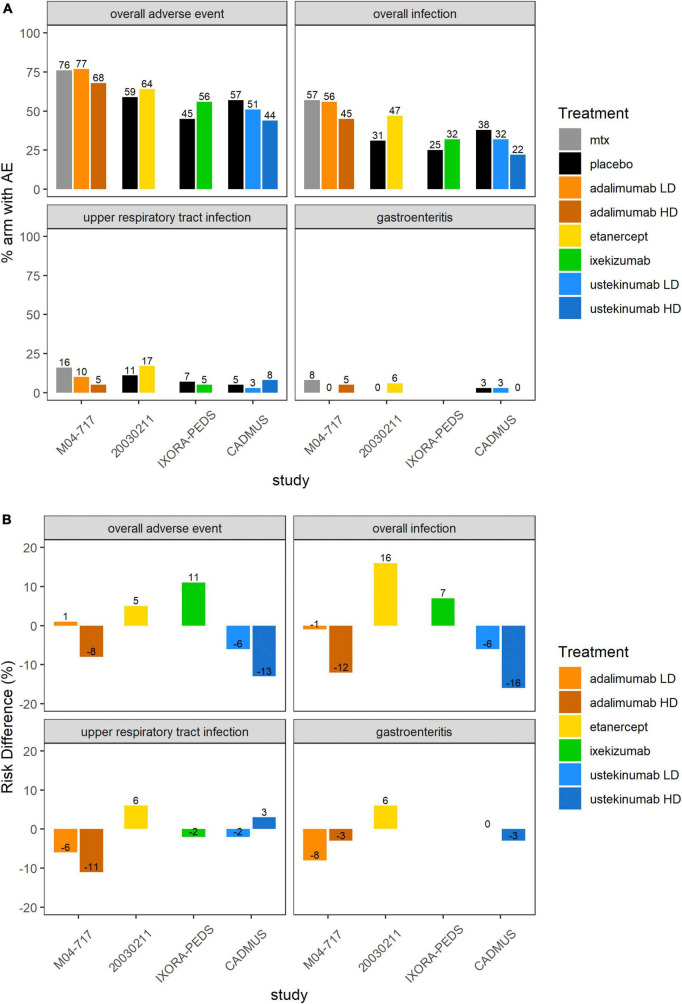
Overall AEs, overall infections, UTRIs or gastroenteritis in the 5 included pediatric psoriasis RCTs. **(A)** Proportion (%) of patients with AEs in each study arm. Higher proportion of AEs in the control arm favors experimental treatment. **(B)** RD (%) of patients with AEs in each treatment arm compared to placebo or SOC. Treatment effect < 0 favors experimental treatment over control arm. AE, adverse event; MTX, methotrexate; RCT, randomized controlled trial; RD, risk difference.

Supplementary Figure 1 and Figure 4 show an overview of the frequency of overall AEs, overall infections, URTIs and gastroenteritis in pediatric plaque psoriasis studies with the exception of the secukinumab studies. There was limited safety data for the induction phase of the CAIN457A2310 study, and hence this study was not included in this analysis. The incidence of overall AEs, overall infections, and URTIs appeared to be more frequent with TNF inhibitors etanercept (64, 47, and 17% respectively), adalimumab 0.4 mg/kg (77, 56, and 10%, respectively) and adalimumab 0.8 mg/kg (68, 45, and 5%, respectively) than with the IL-12/23 ustekinumab 0.375 mg/kg (51, 32, and 3%, respectively) or ustekinumab 0.75 mg/kg (44, 22, and 8%, respectively) (Figure 4). In the pediatric IL-17 studies, frequencies of Candida infections were 0–1.8%, for inflammatory bowel disease (IBD) 0–1.5%. The relative incidence of overall AEs and overall infections was higher with etanercept (64 versus 59% and 47 versus 31%, respectively) (Supplementary Figure 1 and Table 4) and ixekizumab (56 versus 45% and 32 versus 25%, respectively) compared to placebo arms. Of other AEs of interest, airway infections such as nasopharyngitis, pharyngitis, sinusitis and URTI were the most frequently reported (Table 4). Injection-site reactions were reported in 14 patients in the ixekinumab arm versus 1 patient in the control arm in the IXORA-PEDS trial. Overall, no unexpected safety outcomes were found.

#### Adverse events in adults

The incidence of overall AEs, SAEs, overall infections and URTIs in adult psoriasis studies irrespective of dose is shown in Supplementary Table 3. It should be noted that two ustekinumab studies did not report any safety data. Ten other studies did not report safety data at the end of induction (i.e., after 12 and 16 weeks of treatment) and thus were not included in the analysis. Overall AEs were higher in bordalumab, ixekizumab, and secukinumab (58, 59, and 61%, respectively) than in guselkumab, risankizumab, and tildrakizumab (51, 48, and 49%, respectively). Overall infections had higher incidences in certolizumab and secukinumab (37 and 30%, respectively) than in guselkumab and risankizumab (24 and 22%, respectively). Overall infections were not reported in tildrakizumab studies. Incidence of gastroenteritis was low (up to 3%) in all study arms. URTIs were more prevalent in adalimumab, etanercept, and guselkumab (7, 6, and 6%, respectively) than in ixekizumab, secukinumab, and tildrakizumab (4, 3, and 2%, respectively). In general, the relative incidence of overall AEs and overall infections was higher in ixekizumab (59 versus 47% and 27 versus 23%, respectively) and secukinumab (61 versus 53% and 30 versus 20%, respectively) than in placebo arms. The IL-17 drug class had higher risk (RR = 1.16) and the IL-23 drug class had lower risk (RR = 0.97) for overall AEs. In general, SAE incidences in investigated RCTs in adult psoriasis were low with low relative risk (RR = 1.08 for IL-17 drug class, and RR = 1.09 for IL-23 drug class).

#### Anti-drug antibodies in children

There were no anti-drug antibodies reported in RCTs in children with psoriasis (Table 4).

#### Anti-drug antibodies in adults

There was limited information on anti-drug antibodies in RCTs in adults with psoriasis. Administration of adalimumab and ixekizumab (9% for both) was found to have higher incidences of anti-drug antibodies than other investigated biologics (Supplementary Table 3).

### Approved doses of biologics in pediatric and adult psoriasis

Table 5 provides an overview of adult and pediatric doses of biologics as approved by the Food and Drug Administration (FDA) and European Medicines Agency (EMA) (February 3, 2022). All listed drugs are approved by both the FDA and EMA with the exception of adalimumab (approved for pediatric psoriasis by the EMA but not the FDA). Etanercept is approved for psoriasis in children ≥6 years by the EMA and ≥4 years by the FDA but all other biologics are approved for psoriasis in children ≥6 years.

Adalimumab (TNF inhibitor), etanercept (TNF inhibitor), and ustekinumab (IL-12/23 inhibitor) are approved for both adult and pediatric psoriasis. Certolizmab pegol and infliximab are approved for adults but not children. Of the IL-17 inhibitors, ixekizumab and secukinumab are approved for both adult and pediatric populations but brodalumab is only approved in adults (since 2017). IL-23 inhibitors guselkumab, risankizumab and tildakizumab are also only approved for adults (approved between 2017 and 2019). There are ongoing studies of certolizumab (NCT04123795), brodalumab (NCT04305327), guselkumab (NCT03451851), risankizumab (NCT04435600), and tildakizumab (NCT03997786) in pediatric psoriasis patients. On average, biologics in children are approved 4–8 years after approval in adults with plaque psoriasis (Table 5).

As compared to adult doses, children with psoriasis are dosed primarily by weight. Dosing for ixekizumab and secukinumab in children is based on a threshold of 50 kg. Secukinumab is approved for children under 25 kg by the FDA but not the EMA. The dosing algorithm for ustekinumab is similar for adults and children with the exception of children under 60 kg, where it is adjusted by weight (0.75 mg/kg). Besides ustekinumab, dosing of approved biologics in adults with plaque psoriasis is not dependent on weight (Table 5).

## Discussion

To the best of our knowledge, this is the first integrated study that analyzes and compares the efficacy and safety balance of biologics in children and adults with psoriasis. The study by Sun et al. performed a systematic review and meta-analysis of five RCTs assessing biologic therapy in pediatric patients with psoriasis and showed a high efficacy and safety profile (9). In the present work, we summarize and compare efficacy and safety outcomes in children and adults with moderate-to-severe psoriasis, and provide an overview of approved doses in pediatric and adult psoriasis to better understand the efficacy-safety balance of biologics, especially in pediatrics, and to be aware of what we know and do not know about the efficacy-safety biologics in children with moderate-to-severe psoriasis. In addition to PASI75 and PASI90 scores, we summarize Physician’s Global Assessment of Disease Activity (PGA) and Children’s Dermatology Life Quality Index (CDLQI), bringing additional insights into associations between disease progression and health-related quality of life and socioeconomic impacts on patients’ lives.

### Comparing efficacy outcomes in children and adults with psoriasis

PASI75 indicates a 75% or greater reduction in PASI scores from baseline and is indicative of excellent disease improvement. Investigated TNF inhibitors (adalimumab and etanercept) and IL-17 and IL-12/23 inhibitors (ixekizumab, secukinumab, and ustekinumab) evaluated in pediatric plaque psoriasis achieve superior efficacy over control arms, measured by PASI75 and PASI90 after 3 months of treatment. For PASI75, secukinumab and ustekinumab had the highest RR, whereas the TNF inhibitor adalimumab had the lowest RR in studies with pediatric psoriasis. For PASI90, ixekizumab and secukinumab had the highest RR, whereas the TNF inhibitor etanercept had the lowest RR in studies with pediatric psoriasis. For the adult studies, ixekizumab and secukinumab had the highest pooled RR for PASI75 and PASI90. Achieving a PGA score of 0 (cleared) to 1 (minimal) also indicates extensive reduction in disease burden. Ustekinumab and ixekizumab led to the highest percentages of patients achieving a PGA score of 0 or 1 after 3 months of treatment. Treatment with ixekizumab, etanercept and secukinumab high-dose showed the most significant effect on percent improvement from baseline in CDLQI after 3 months of treatment, therefore accounting for a higher impact on psychosocial health.

### Comparing safety outcomes in children and adults with psoriasis

Overall, no unexpected safety outcomes were found in this systematic literature review and psoriasis treatment with biologics appears to be safe. Incidences of overall AEs, overall infections, and URTIs were more frequent with the TNF inhibitors etanercept and adalimumab than with ustekinumab. The relative incidence of overall AEs and overall infection was higher in etanercept and ixekizumab compared to placebo arms. Literature suggests that children might be slightly less prone to Candida infection with IL-17 treatment (72). A clear association of IBD and IL-17 treatment could not be corroborated in adults and pediatric studies tend to support this conclusion (42). Overall, etanercept administration yielded more infections than in the control group. SAE incidences with biologics were low (up to 3%) in investigated pediatric and adult psoriasis RCTs, except for a pediatric study arm with the TNF inhibitor adalimumab (8%). This finding is also reflected in the literature of adult patients and seems to mirror a real effect of a higher risk with anti-TNF as compared to the newer classes of biologics (73). The aims of the present study were to compare and review efficacy and safety data at 12 weeks, which is the standard timepoint for the pivotal trials leading to approval. Long-term safety outcomes could therefore not be assessed beyond 2 years in this study as investigated RCTs reported safety data up to 2 years only (74). It should be noted that several prior registries have focused on long-term observations in children treated with biologics (75, 76).

Overall, the risk difference (RD) of overall infections, overall AEs, and URTIs was similar between adults and children with psoriasis. A recent meta-analysis on plaque psoriasis treated with biologic drugs performed by Cui et al. showed a similar relative risk of overall AEs and SAEs in comparison to our report (77). In general, study arms with biologics did not have considerably higher short-term safety concerns than control arms. Additional clinical trials in pediatric psoriasis patients are warranted to further characterize longer-term safety outcomes and bring additional evidence to the use of this new class of biologics in this particularly vulnerable patient population.

### Comparing dosing of approved biologics in children and adults with psoriasis

Besides ustekinumab and etanercept, dosing of approved biologics in adults with plaque psoriasis is not dependent on weight. In contrast, children with psoriasis are dosed primarily by weight. Dosing for ixekizumab and secukinumab in children is based on a threshold of 50 kg. Secukinumab is approved for children under 25 kg by the FDA but not the EMA. The dosing algorithm for ustekinumab is similar for adults and children with the exception of children under 60 kg, whereas dosing of secukinumab in children corresponds to less than half the adult dose. Dosing regimens of etanercept and ixekizumab in adults and children are similar, although administration tends to be less frequent in children as compared to adults with psoriasis.

### Use and development of biologics in pediatric psoriasis

The use of biologics in pediatric dermatology has strongly increased within the last few years and treatment guidelines specifically addressing pediatric psoriasis have been published (8). The therapeutic armamentarium has never been bigger and is still growing, enabling effective treatment for the large majority of children with psoriasis. Given the range of excellent medications, treatment decisions rely on safety and efficacy, comorbidities, patient/doctor preference such as frequency of application, as well as local regulations. Given the overall safety and efficacy data investigated in this systematic literature review and meta-analysis, the newer IL12/23- and IL17-targeting biologics seem preferable to the older TNF inhibitors for a majority of pediatric patients with moderate-to-severe psoriasis. This is in line with other reports explaining that monoclonal antibodies inhibiting IL-17 signaling (secukinumab, brodalumab and ixekizumab) and newer IL-23 antagonists (guselkumab, tildrakizumab and risankizumab) may offer greater disease control in psoriasis by acting on the main cytokine pathways driving psoriatic disease (3).

On average, biologics in children are approved 4 to 8 years after approval in adults with psoriasis. An enhanced understanding of the efficacy-safety balance of existing and new biologics will facilitate decision-making in clinical practice as well as the development of new treatment options in children with psoriasis. Exploratory meta-analyses and model-based meta-analysis can utilize integrated data from clinical controlled studies strengthening the knowledge of a particular drug and its efficacy and safety relative to other treatment options in adults and children (78, 79). Meta-analysis, especially model-based meta-analysis, facilitates the bridging of clinical outcomes data from adult and pediatric patients and can be a useful quantitative tool for enhancing key decisions such as dose selection and study design (Pediatric Investigation Plan, PIP), product positioning, and go/no-go decisions in pediatric drug development (80–82). To the best of our knowledge this is the first study that describes and compares efficacy and safety profiles of biologics currently prescribed in adults and children with moderate-to-severe psoriasis.

### Limitations

Very limited pediatric data exist compared to adult data in the evaluation of biologics in plaque psoriasis. Nevertheless, RCTs included in this study showed high-quality data and strong RCT selection criteria were used while performing the literature search. There are several limitations to this systematic literature review, including the relatively small number of pediatric studies and number of therapeutic entities reported in previously published RCTs. First, the focus of this analysis was to primarily compare the efficacy of biologics based on the primary endpoints PASI75 and PASI90 3 months after treatment initiation in pediatric and adult psoriasis patients. It should also be noted that in one pediatric RCT (CADMUS) severe psoriasis was set as inclusion criterion as compared to moderate-to-severe psoriasis in all other pediatric RCTs. Longer-term efficacy and safety data were not available from all trials included in the analysis due to shorter treatment duration, or they were available but at different timepoints across trials. The studies’ inclusion criterion for efficacy and safety assessment was set at 12 weeks because the typical timepoint for efficacy and safety evaluation for the pivotal trials leading to drug approval is 12 weeks. We therefore interpret reported safety data with caution and are aware that additional longer-term data are warranted to fully understand the overall efficacy-safety profile of biologics in children with moderate-to-severe psoriasis.

## Conclusion

Both adults and children with psoriasis showed superior efficacy responses with biologics compared to placebo or SOC after 3 months of treatment, with SAE incidences in the low percentages compared to control arms. Monoclonal antibodies inhibiting IL-17 signaling and newer IL-12/23 antagonists may offer even greater disease control in pediatric psoriasis with similar or fewer SAEs than TNF-inhibitors. The performed meta-analysis comparing clinical outcomes in children and adults can further enhance understanding of the efficacy-safety balance of biologics in pediatric psoriasis. Additional clinical studies are warranted to better characterize longer-term safety outcomes of biologics including newer ones in children with moderate-to-severe psoriasis.

## Author contributions

KG, CW, and Y-HY performed the systematic review of efficacy and safety data. TW was involved in setting up the systemic literature search. KG and CW were responsible for execution and documentation. MT provided support as therapeutic area expert. Any discrepancies were resolved through discussion or consultations with a third independent reviewer MP. KG, CW, MT, MB, Y-HY, NZ, and MP have contributed in the preparation of the submitted manuscript, were involved in designing and critically revising the research project, have approved this version to be published and they agreed to be accountable for all aspects in the work in ensuring questions related to the accuracy or integrity of any part of the work appropriately investigated and resolved, and have agreed to the submission of this manuscript to Frontiers. All authors contributed to the article and approved the submitted version.

## Footnotes

1
ClinicalTrials.gov

## Supplementary material

Supplementary Figure 1 Comparing efficacy (PASI75 RRs) against safety (RD) in the RCTs that reported both outcomes. **(A)** PASI75 RR versus overall AE RD; **(B)** PASI75 RR versus overall infection RD. AE, adverse event; HD, high dose; LD, low dose; PASI, Psoriasis Area and Severity Index; RCT, randomized controlled trial; RD, risk difference.

Supplementary Figure 2 Funnel plots asymmetry tests, using data from pediatric psoriasis studies **(A)** PASI75 data **(B)** PASI90 data, with log-risk ratios displayed on the horizontal axis. PASI, Psoriasis Area and Severity Index.
