# Supplementary material for: Understanding efficacy-safety balance of biologics in moderate-to-severe pediatric psoriasis
Source: Front Med (Lausanne). 2022 Sep 26;9:944208. doi: 10.3389/fmed.2022.944208 (PMC9548699; doi:10.3389/fmed.2022.944208)
Supplement: Supplementary file 1 [file Table_1.pdf]

Drug class	Drug	Endpoint	Ndrug	Nplacebo	Ntotal	#Studies	#Study arms	Risk Ratio (RR)	RR CI Low	RR CI High
TNF inhibitor	adalimumab	PASI75	1855	852	2707	7	10	11.8	7.86	17.72
		PASI90	1760	800	2560	6	8	19.68	12.26	31.6
	certolizumab	PASI75	810	216	1026	4	8	13.45	7.73	23.4
		PASI90	810	216	1026	4	8	28.8	9.27	89.45
	etanercept	PASI75	3607	2016	5623	14	17	8.79	7.35	10.52
		PASI90	3607	2016	5623	14	17	11.65	7.56	17.94
IL-17 inhibitor	brodalumab	PASI75	3249	947	4196	7	17	12.66	9.58	16.74
		PASI90	3249	947	4196	7	17	26.82	18.1	39.73
	ixekizumab	PASI75	2449	818	3267	4	10	16.18	11.83	22.14
		PASI90	2449	818	3267	4	10	46.49	23.66	91.36
	secukinumab	PASI75	2569	1112	3681	13	26	15.35	12.49	18.86
		PASI90	2547	1140	3687	14	24	29.18	20.6	41.35
IL-12/23 inhibitor	ustekinumab	PASI75	2779	1866	4645	12	18	12.65	9.73	16.45
		PASI90	2768	1853	4621	11	17	22.48	16.71	30.24
IL-23 inhibitor	guselkumab	PASI75	920	478	1398	5	10	23.02	7.7	68.83
		PASI90*	463	240	703	3	7	NA	NA	NA
	risankizumab	PASI75	642	208	850	3	4	9.92	6.45	15.26
		PASI90	642	208	850	3	4	21.85	9.91	48.17
	tildrakizumab	PASI75	1545	355	1900	3	8	10.97	8.2	14.67
		PASI90	1238	311	1549	2	4	17.55	9.97	30.89

Supplementary Table 1. Overview of PASI75/90 data at three months pooled across RCTs in adults with psoriasis

Abbreviations: CI, confidence interval; NA, not available; Ndrug, number of patients receiving the drug; Nplacebo, number of patients receiving the placebo; Ntotal, total number of patients; PASI, Psoriasis Area and Severity Index; RCT, randomized controlled trial; RR, risk ratio

* NA as RRs were Inf due to PASI90 response of 0% in placebo arm of almost all studies
