# Supplementary material for: Understanding efficacy-safety balance of biologics in moderate-to-severe pediatric psoriasis
Source: Front Med (Lausanne). 2022 Sep 26;9:944208. doi: 10.3389/fmed.2022.944208 (PMC9548699; doi:10.3389/fmed.2022.944208)
Supplement: Supplementary file 2 [file Table_2.DOCX]

| **Drug Class** | **Drug** | **Endpoint** | **Ndrug** | **Nplacebo** | **Ntotal** | **#Studies** | **#Study arms** | **Risk Ratio (RR)** | **RR CI Low** | **RR CI High** |
| --- | --- | --- | --- | --- | --- | --- | --- | --- | --- | --- |
| TNF inhibitor | adalimumab | DLQI ≤1 | 338 | 87 | 425 | 1 | 1 | 2.76 | 1.45 | 5.24 |
|  |  | PGA ≤1 | 1303 | 580 | 1883 | 4 | 4 | 10.37 | 5.62 | 19.12 |
|  | certolizumab | DLQI ≤1 | 104 | 40 | 144 | 1 | 2 | 4.16 | 2.42 | 7.16 |
|  |  | PGA ≤1 | 810 | 216 | 1026 | 4 | 8 | 30.47 | 11.48 | 80.86 |
|  | etanercept | DLQI ≤1 | 1475 | 665 | 2140 | 5 | 5 | 4.88 | 3.71 | 6.43 |
|  |  | PGA ≤1 | 2926 | 1499 | 4425 | 11 | 14 | 7.74 | 5.83 | 10.27 |
| IL-17 inhibitor | brodalumab | DLQI ≤1 | 158 | 37 | 195 | 1 | 4 | 9.65 | 4.82 | 19.31 |
|  |  | PGA ≤1 | 3249 | 947 | 4196 | 7 | 17 | 17.95 | 14.18 | 22.73 |
|  | ixekizumab | DLQI ≤1 | 1469 | 361 | 1830 | 2 | 4 | 9.06 | 6.91 | 11.86 |
|  |  | PGA ≤1 | 2449 | 818 | 3267 | 4 | 10 | 15.84 | 11.05 | 22.7 |
|  | secukinumab | DLQI ≤1 | 1055 | 427 | 1482 | 4 | 9 | 5.45 | 4.37 | 6.8 |
|  |  | PGA ≤1 | 2566 | 1039 | 3605 | 10 | 25 | 14.54 | 9.2 | 22.98 |
| IL-12/23 inhibitor | ustekinumab | DLQI ≤1 | 1488 | 742 | 2230 | 4 | 7 | 12.23 | 8.72 | 17.15 |
|  |  | PGA ≤1 | 2746 | 1806 | 4552 | 10 | 16 | 11.31 | 8.71 | 14.69 |
| IL-23 inhibitor | guselkumab | DLQI ≤1 | 0 | 0 | 0 | 0 | 0 | NA | NA | NA |
|  |  | PGA ≤1 | 591 | 304 | 895 | 4 | 9 | 13.65 | 5.42 | 34.39 |
|  | risankizumab | DLQI ≤1 | 0 | 0 | 0 | 0 | 0 | NA | NA | NA |
|  |  | PGA ≤1 | 642 | 208 | 850 | 3 | 4 | 9.4 | 6.11 | 14.46 |
|  | tildrakizumab | DLQI ≤1 | 1224 | 306 | 1530 | 2 | 4 | 6.4 | 4.71 | 8.69 |
|  |  | PGA ≤1 | 1238 | 310 | 1548 | 2 | 4 | 9.27 | 6.79 | 12.65 |

**Supplementary Table 2. Overview of PGA and DLQI data at three months in RCTs in adults with psoriasis**

*Abbreviations:* CI, confidence interval; DLQI, Dermatology Life Quality Index; ; Ndrug, number of patients receiving the drug; Nplacebo, number of patients receiving the placebo; Ntotal, total number of patients; PGA, Physician Global Assessment; RCT, randomized controlled trial; RR, risk ratio
