# Supplementary material for: Understanding efficacy-safety balance of biologics in moderate-to-severe pediatric psoriasis
Source: Front Med (Lausanne). 2022 Sep 26;9:944208. doi: 10.3389/fmed.2022.944208 (PMC9548699; doi:10.3389/fmed.2022.944208)
Supplement: Supplementary file 3 [file Table_3.DOCX]

|  |  | **AE any** |  |  |  |  | **AE serious** | |  |  |  | **Infection Any** | | |  |  | **URTI** |  |  |  |  |
| --- | --- | --- | --- | --- | --- | --- | --- | --- | --- | --- | --- | --- | --- | --- | --- | --- | --- | --- | --- | --- | --- |
|  |  | **Biologics** |  | **Placebo** |  |  | **Biologics** | | **Placebo** |  |  | **Biologics** |  | **Placebo** |  |  | **Biologics** |  | **Placebo** |  |  |
| **Drug class** | **Drug** | **n/N** | **%** | **n/N** | **%** | **RD** | **n/N** | **%** | **n/N** | **%** | **RD** | **n/N** | **%** | **n/N** | **%** | **RD** | **n/N** | **%** | **n/N** | **%** | **RD** |
| TNF inhibitor | adalimumab | 1004/1730 | 58 | 439/806 | 54 | 4 | 33/1730 | 2 | 15/806 | 2 | 0 | 435/1635 | 27 | 176/754 | 23 | 3 | 111/1528 | 7 | 29/659 | 4 | 3 |
|  | certolizumab | 466/809 | 58 | 134/215 | 62 | -5 | 23/809 | 3 | 7/215 | 3 | 0 | 43/117 | 37 | 24/58 | 41 | -5 | 38/692 | 5 | 11/157 | 7 | -2 |
|  | etanercept | 1011/1866 | 54 | 605/1213 | 50 | 4 | 41/2671 | 2 | 33/1605 | 2 | -1 | 524/2094 | 25 | 283/1346 | 21 | 4 | 213/3547 | 6 | 102/1964 | 5 | 1 |
| IL-12/23 inhibitor | ustekinumab | 1327/2489 | 53 | 866/1740 | 50 | 4 | 40/2489 | 2 | 30/1740 | 2 | 0 | 455/1876 | 24 | 227/1118 | 20 | 4 | 127/2489 | 5 | 89/1740 | 5 | 0 |
| IL-17 inhibitor | brodalumab | 1882/3241 | 58 | 484/945 | 51 | 7 | 53/3201 | 2 | 16/923 | 2 | 0 | 0/20 | 0 | 2/5 | 40 | -40 | 167/3241 | 5 | 59/945 | 6 | -1 |
|  | ixekizumab | 1364/2328 | 59 | 379/791 | 47 | 11 | 46/2328 | 2 | 12/791 | 2 | 0 | 633/2328 | 27 | 181/791 | 23 | 4 | 96/2328 | 4 | 28/791 | 4 | 1 |
|  | secukinumab | 1182/1942 | 61 | 448/850 | 53 | 8 | 47/2085 | 2 | 19/921 | 2 | 0 | 446/1498 | 30 | 132/659 | 20 | 10 | 62/2067 | 3 | 14/903 | 2 | 1 |
| IL-23 inhibitor | guselkumab | 370/726 | 51 | 156/296 | 53 | -2 | 15/726 | 2 | 6/296 | 2 | 0 | 159/664 | 24 | 64/280 | 23 | 1 | 38/640 | 6 | 9/190 | 5 | 1 |
|  | risankizumab | 285/598 | 48 | 97/200 | 49 | -1 | 13/598 | 2 | 4/200 | 2 | 0 | 131/598 | 22 | 26/200 | 13 | 9 | 28/598 | 5 | 4/200 | 2 | 3 |
|  | tildrakizumab | 765/1546 | 49 | 191/355 | 54 | -4 | 27/1546 | 2 | 5/355 | 1 | 0 | NA | NA | NA | NA | NA | 32/1546 | 2 | 9/355 | 3 | 0 |

**Supplementary Table 3. Overview of pooled safety data in adult psoriasis RCTs**

*Abbreviations:* AE, adverse event; RD, risk difference; URTI, upper respiratory tract infection; NA, not available
