# Supplementary figures and images for: Understanding efficacy-safety balance of biologics in moderate-to-severe pediatric psoriasis
Source: Front Med (Lausanne). 2022 Sep 26;9:944208. doi: 10.3389/fmed.2022.944208 (PMC9548699; doi:10.3389/fmed.2022.944208)

Figure 1A


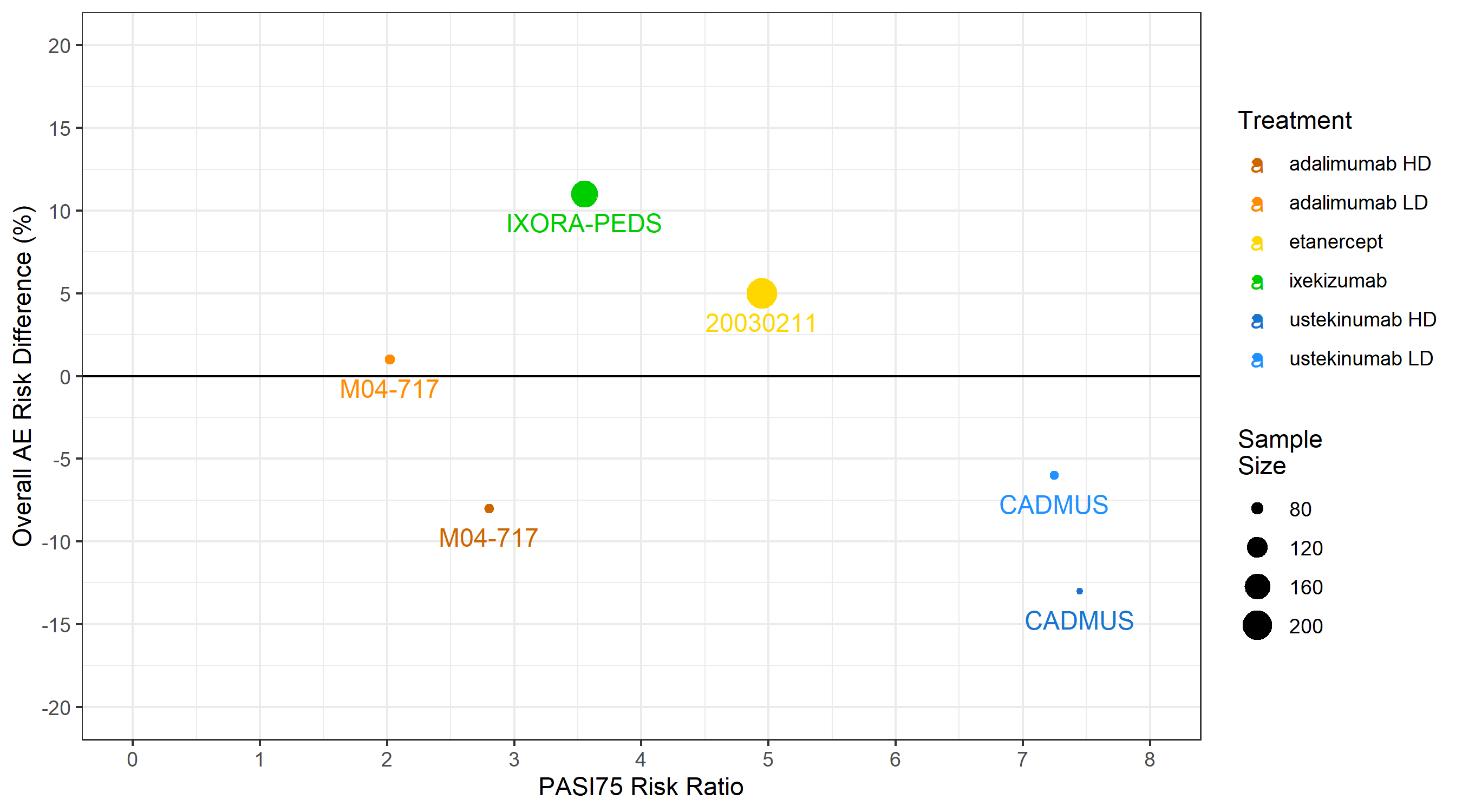


Figure 1B


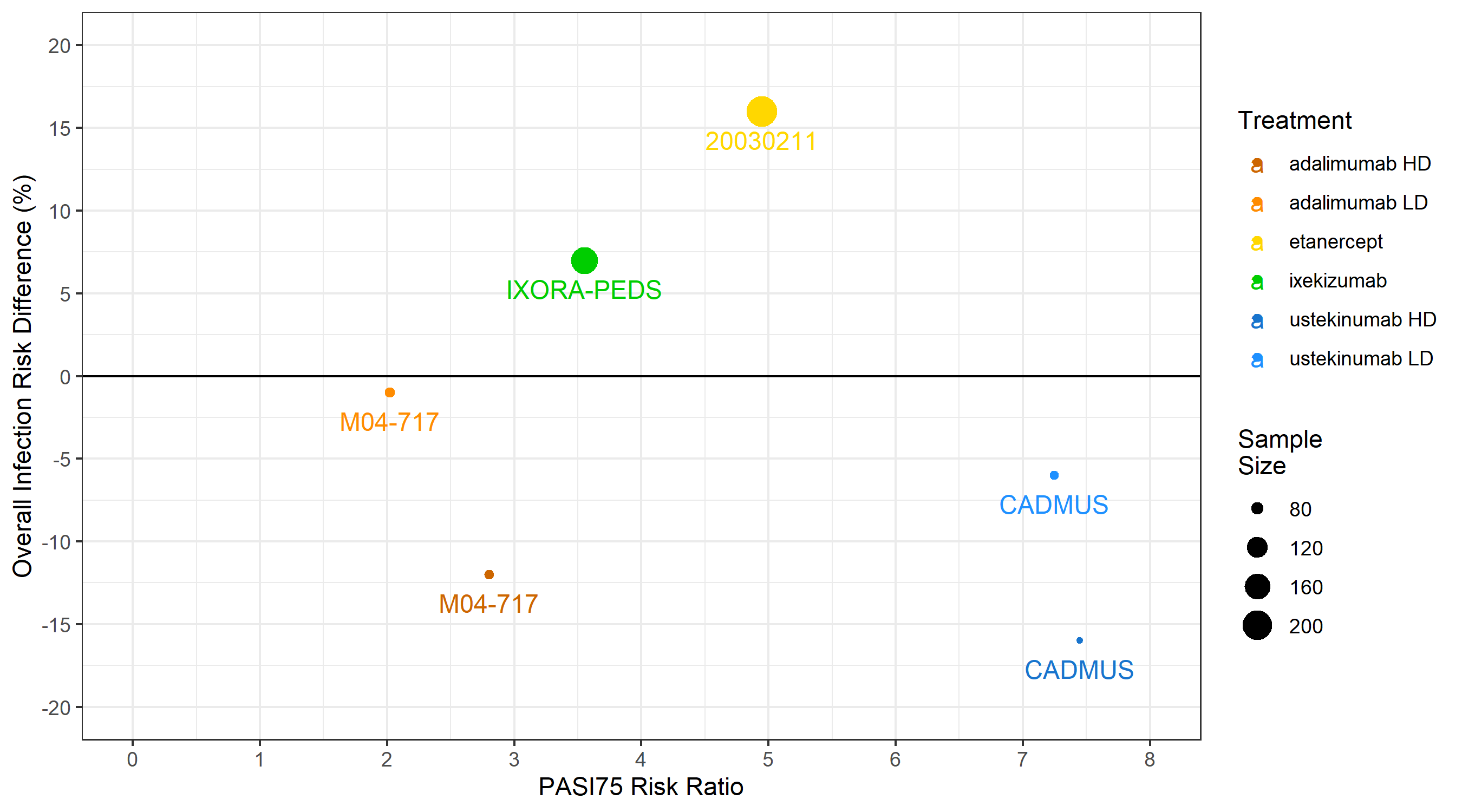

Supplement: Supplementary Figure 1 — Comparing efficacy (PASI75 RRs) against safety (RD) in the RCTs that reported both outcomes. (A) PASI75 RR versus overall AE RD; (B) PASI75 RR versus overall infection RD. AE, adverse event; HD, high dose; LD, low dose; PASI, Psoriasis Area and Severity Index; RCT, randomized controlled trial; RD, risk difference. [file Image_1.docx]

Figure 2A
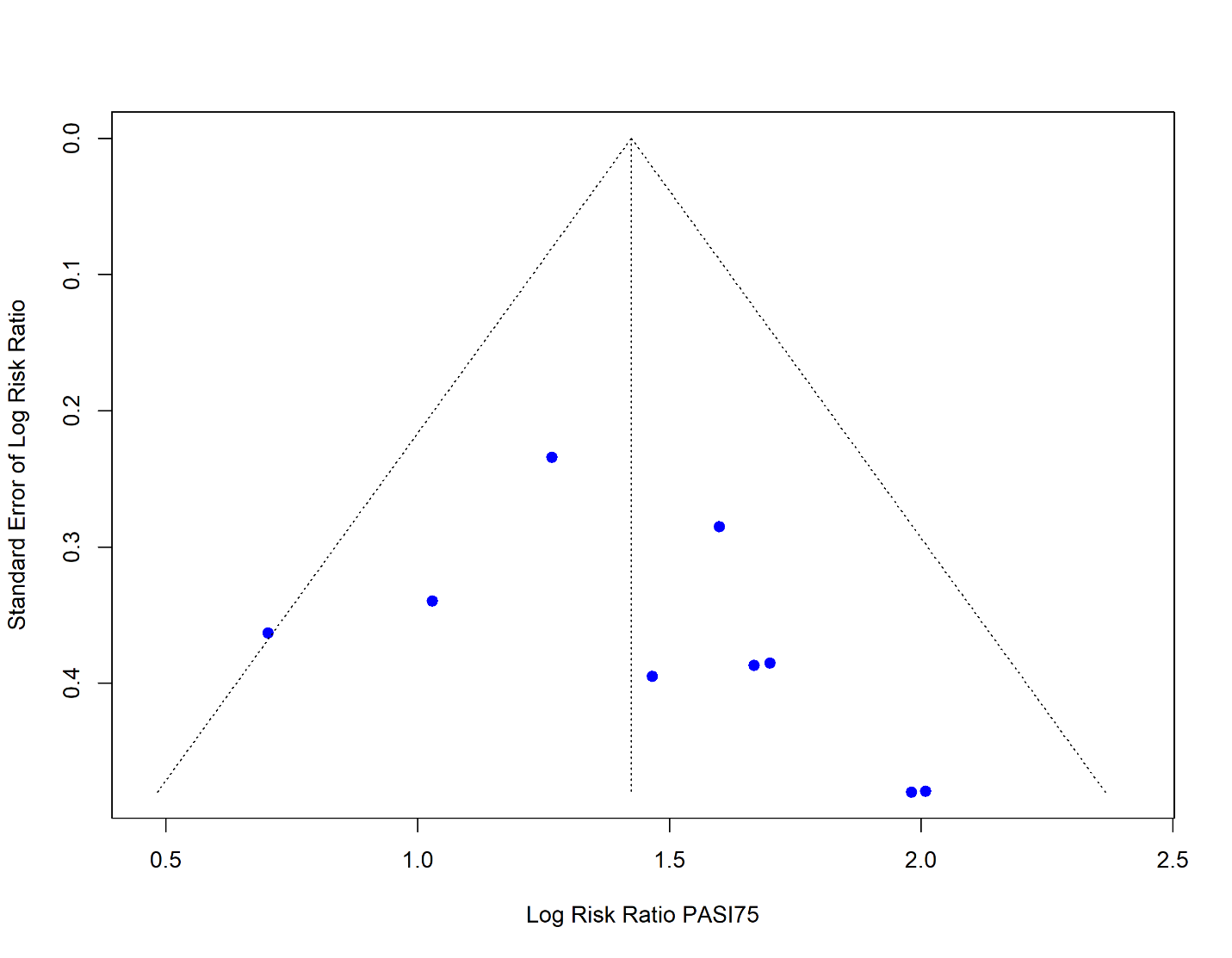


Figure 2B
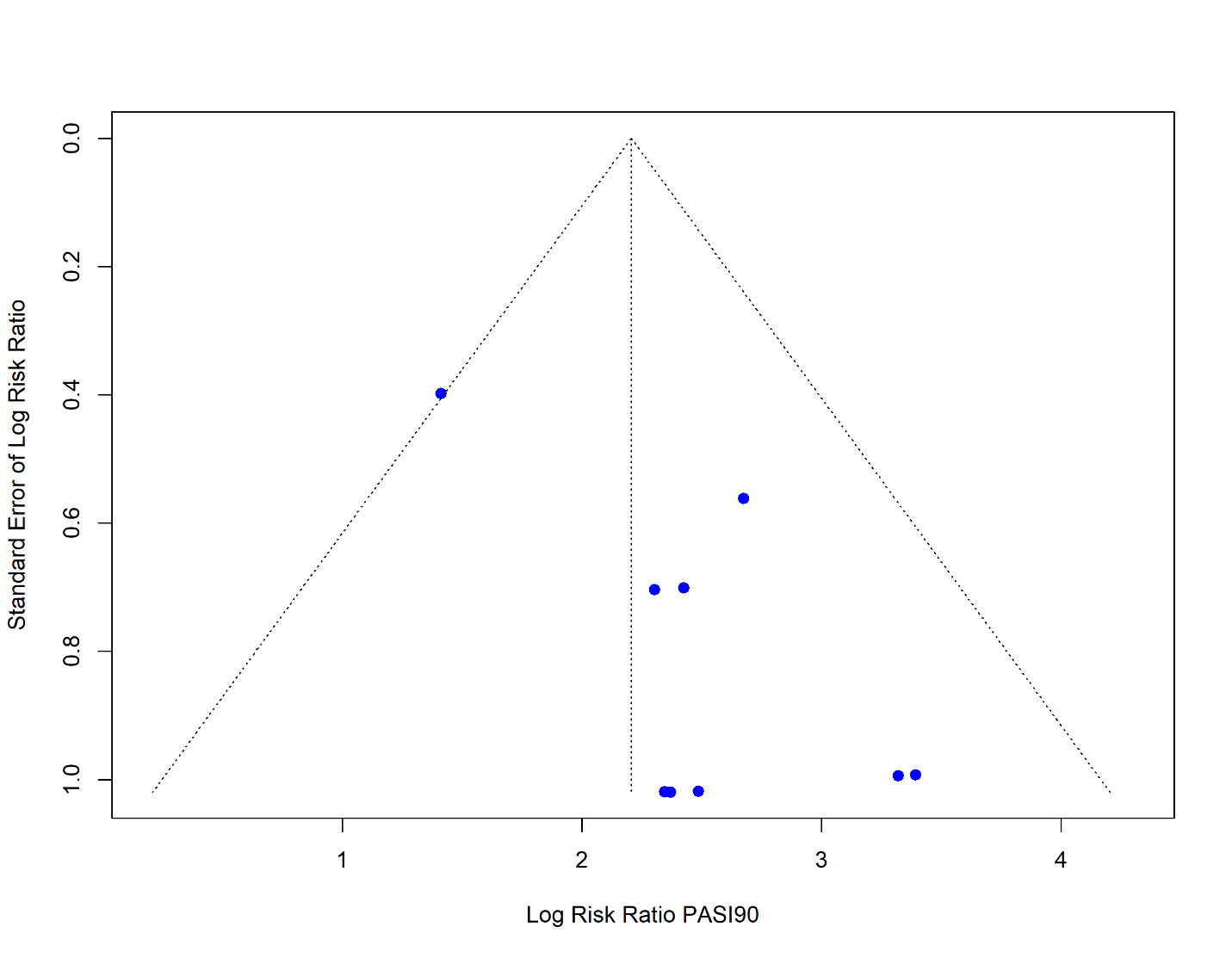

Supplement: Supplementary Figure 2 — Funnel plots asymmetry tests, using data from pediatric psoriasis studies (A) PASI75 data (B) PASI90 data, with log-risk ratios displayed on the horizontal axis. PASI, Psoriasis Area and Severity Index. [file Image_2.docx]
